# Association of Medicaid Expansion With Opioid Overdose Mortality in the United States

**DOI:** 10.1001/jamanetworkopen.2019.19066

**Published:** 2020-01-10

**Authors:** Nicole Kravitz-Wirtz, Corey S. Davis, William R. Ponicki, Ariadne Rivera-Aguirre, Brandon D. L. Marshall, Silvia S. Martins, Magdalena Cerdá

**Affiliations:** 1 Violence Prevention Research Program, Department of Emergency Medicine, University of California Davis School of Medicine, Sacramento; 2 Network for Public Health Law, Los Angeles, California; 3 Prevention Research Center, Pacific Institute for Research and Evaluation, Berkeley, California; 4 Center for Opioid Epidemiology and Policy, Department of Population Health, New York University School of Medicine, New York; 5 Department of Epidemiology, Brown University School of Public Health, Providence, Rhode Island; 6 Mailman School of Public Health, Department of Epidemiology, Columbia University, New York, New York

## Abstract

**Importance:**

The Patient Protection and Affordable Care Act (ACA) permits states to expand Medicaid coverage for most low-income adults to 138% of the federal poverty level and requires the provision of mental health and substance use disorder services on parity with other medical and surgical services. Uptake of substance use disorder services with medications for opioid use disorder has increased more in Medicaid expansion states than in nonexpansion states, but whether ACA-related Medicaid expansion is associated with county-level opioid overdose mortality has not been examined.

**Objective:**

To examine whether Medicaid expansion is associated with county × year counts of opioid overdose deaths overall and by class of opioid.

**Design, Setting, and Participants:**

This serial cross-sectional study used data from 3109 counties within 49 states and the District of Columbia from January 1, 2001, to December 31, 2017 (N = 3109 counties × 17 years = 52 853 county-years). Overdose deaths were modeled using hierarchical Bayesian Poisson models. Analyses were performed from April 1, 2018, to July 31, 2019.

**Exposures:**

The primary exposure was state adoption of Medicaid expansion under the ACA, measured as the proportion of each calendar year during which a given state had Medicaid expansion in effect. By the end of study observation in 2017, a total of 32 states and the District of Columbia had expanded Medicaid eligibility.

**Main Outcomes and Measures:**

The outcomes of interest were annual county-level mortality from overdoses involving any opioid, natural and semisynthetic opioids, methadone, heroin, and synthetic opioids other than methadone, derived from the National Vital Statistics System multiple-cause-of-death files. A secondary analysis examined fatal overdoses involving all drugs.

**Results:**

There were 383 091 opioid overdose fatalities across observed US counties during the study period, with a mean (SD) of 7.25 (27.45) deaths per county (range, 0-1145 deaths per county). Adoption of Medicaid expansion was associated with a 6% lower rate of total opioid overdose deaths compared with the rate in nonexpansion states (relative rate [RR], 0.94; 95% credible interval [CrI], 0.91-0.98). Counties in expansion states had an 11% lower rate of death involving heroin (RR, 0.89; 95% CrI, 0.84-0.94) and a 10% lower rate of death involving synthetic opioids other than methadone (RR, 0.90; 95% CrI, 0.84-0.96) compared with counties in nonexpansion states. An 11% increase was observed in methadone-related overdose mortality in expansion states (RR, 1.11; 95% CrI, 1.04-1.19). An association between Medicaid expansion and deaths involving natural and semisynthetic opioids was not well supported (RR, 1.03; 95% CrI, 0.98-1.08).

**Conclusions and Relevance:**

Medicaid expansion was associated with reductions in total opioid overdose deaths, particularly deaths involving heroin and synthetic opioids other than methadone, but increases in methadone-related mortality. As states invest more resources in addressing the opioid overdose epidemic, attention should be paid to the role that Medicaid expansion may play in reducing opioid overdose mortality, in part through greater access to medications for opioid use disorder.

## Key Points

**Question:**

Is state Medicaid expansion associated with county-level opioid-involved overdose deaths in the United States?

**Findings:**

In this serial cross-sectional study of 3109 counties within 49 states and the District of Columbia from 2001 to 2017, Medicaid expansion was associated with reductions in total opioid overdose deaths and deaths involving heroin and synthetic opioids other than methadone. Expansion was associated with increased mortality involving methadone.

**Meaning:**

The findings suggest that expanding eligibility for Medicaid may help to mitigate the opioid overdose epidemic.

## Teaser

This serial cross-sectional study examines whether US state Medicaid expansion is associated with county-level counts of opioid overdose deaths.

## Introduction

Drug overdose is a leading cause of injury-related death in the United States, responsible for more than 70 000 fatalities, or approximately 200 deaths per day, in 2017. Fatal drug overdoses have increased markedly during the past 2 decades in large part because of overdoses involving opioids, including prescription opioids and illegal opioids, such as heroin and illicitly manufactured fentanyl. Between 2001 and 2017, the age-adjusted mortality rate for opioid-related overdoses more than quadrupled, from 3.3 to 14.9 per 100 000 standard population. In 2017, more than two-thirds of all drug overdose fatalities (47 600 deaths) involved an opioid.^1^ Although overdose mortality may have stabilized in the past year, rates remain inordinately high.

The 2010 Patient Protection and Affordable Care Act (ACA) was signed into law during the rise in overdose deaths. Designed to increase access to and improve the quality of health insurance coverage, the ACA permits states to expand Medicaid coverage to essentially all non–Medicare-eligible people younger than 65 years with incomes at or below 138% of the federal poverty level ($16 643 for an individual in 2017).^2^ The law also requires that individuals who receive coverage through the expansion be provided with mental health and substance use disorder (SUD) services on parity with other medical and surgical services.^3^ From the beginning of Medicaid expansion in 2014 to the end of study observation in 2017, a total of 32 states and the District of Columbia opted to expand Medicaid eligibility.^4^

Medicaid provides essential health care access to millions of low-income people and, by extension, greater access to low-cost prescription medications, including opioid pain relievers (OPRs). Such increased access to OPRs, particularly among a patient population with higher rates of chronic disease and disability compared with non-Medicaid recipients,^5^ has led some observers to question whether Medicaid expansion will contribute to additional opioid-related harms. To the contrary, recent studies^6,7,8^ have found that although Medicaid expansion was associated with an increased rate of overall Medicaid-reimbursed prescriptions, changes in prescriptions for OPRs before vs after the expansion were not significantly different in expansion vs nonexpansion states.

Furthermore, Medicaid expansion has been an important source of coverage for SUD treatment, including for people with opioid use disorder (OUD). Previous research suggests that uptake of medications for opioid use disorder (MOUDs), including methadone, buprenorphine, and extended-release naltrexone, has increased more in expansion states compared with nonexpansion states.^6,7,8,9,10,11^ These medications (often in combination with counseling and behavioral therapies) have been linked to improvements in treatment retention and OUD remission as well as reductions, in some cases as high as 50%, in all-cause and overdose-related mortality.^12,13^ Medicaid-reimbursed prescriptions for the opioid overdose reversal medication naloxone have also increased significantly more in expansion states compared with nonexpansion states.^14^ Early Medicaid expansions in Arizona, Maine, and New York in 2001 and 2002,^15^ along with more recent expansions in state Medicaid-eligibility thresholds for parents,^16^ have been associated with fewer drug overdose deaths. However, to our knowledge, with only 1 recent exception,^17^ no study has examined the association of ACA-related Medicaid expansion with opioid-related overdose mortality more specifically.

Previous studies^12,16,17^ of the association of Medicaid expansion with fatal overdoses have been conducted at the state level. Although the most appropriate spatial scale for this association remains unclear, state-level analyses may not adequately reflect local (within-state) variation in the level and rate of growth of overdose deaths or differences in policy implementation, such as local disparities in the capacity for or accessibility of SUD treatment. Using overdose mortality and related covariates measured at the county rather than the state level, this study aimed to provide improved estimates of the association between Medicaid expansion under the ACA and fatal opioid-involved overdoses from 2001 to 2017. We examined this association for county × year counts of total opioid overdose deaths and separately by class of opioid (ie, natural and semisynthetic opioids, methadone, heroin, and synthetic opioids other than methadone). For comparison with prior research, we also examined all drug overdose deaths as a secondary outcome.

## Methods

This serial, cross-sectional study used data from 3109 counties in 49 states and the District of Columbia from January 1, 2001, to December 31, 2017. We organized this information into a series of space-time observations, with each observation referring to 1 year of data per county for a total of 52 853 county-years (3109 counties × 17 years). Analyses excluded Alaska because of substantial changes in the size and shape of counties within the state during the study period. Individual data were aggregated to the county level. This study and was approved by the institutional review board of the University of California, Davis. No informed consent was required because this was a retrospective review of existing mortality data. The study followed the Strengthening the Reporting of Observational Studies in Epidemiology (STROBE) reporting guideline.

### Outcome

We determined annual, county-level counts of opioid overdose deaths from the restricted-use version of the National Vital Statistics System multiple-cause-of-death files.^18^ Overdose deaths were identified based on the *International Statistical Classification of Diseases and Related Health Problems, Tenth Revision (ICD-10)* external cause of injury codes X40 to 44 (unintentional), X60 to 64 (suicide), X85 (homicide), and Y10 to 14 (undetermined). Among deaths with drug overdose as the underlying cause, we used the following *ICD-10* specific drug codes to identify our outcomes: all opioids, T40.0-T40.4 and T40.6; natural and semisynthetic opioids, T40.2; methadone, T40.3; heroin, T40.1; and synthetic opioids other than methadone, T40.4. Deaths involving more than 1 class of opioid were included in the counts for each opioid subcategory; thus, opioid subcategories are not mutually exclusive.

### Exposure

Data on state Medicaid expansion status were obtained from the Kaiser Family Foundation.^4^ We created an indicator of the proportion of each calendar year during which a given state had Medicaid expansion in effect; states that expanded Medicaid were assigned a value of 0 in years before Medicaid expansion, a value between 0 and 1 in the year in which Medicaid expansion went into effect (according to the policy effective month), and a value of 1 in all subsequent years, whereas states that did not expand Medicaid by the end of the study period were assigned a value of 0 in all years. Of the 32 states (including the District of Columbia) in our study population that opted to expand Medicaid eligibility, 26 did so on January 1, 2014, then 2 additional states did so later that same year, followed by 2 states in 2015 and 2 states in 2016 (Table 1).

**Table 1.** Status and Effective Date of Medicaid Expansion by State^a^

State	Status	Effective Date
Alabama	Not adopted	NA
Alaska^b^	Adopted	September 1, 2015
Arizona	Adopted	January 1, 2014
Arkansas	Adopted	January 1, 2014
California	Adopted	January 1, 2014
Colorado	Adopted	January 1, 2014
Connecticut	Adopted	January 1, 2014
Delaware	Adopted	January 1, 2014
District of Columbia	Adopted	January 1, 2014
Florida	Not adopted	NA
Georgia	Not adopted	NA
Hawaii	Adopted	January 1, 2014
Idaho	Not adopted	NA
Illinois	Adopted	January 1, 2014
Indiana	Adopted	February 1, 2015
Iowa	Adopted	January 1, 2014
Kansas	Not adopted	NA
Kentucky	Adopted	January 1, 2014
Louisiana	Adopted	July 1, 2016
Maine	Adopted	January 1, 2014
Maryland	Adopted	January 1, 2014
Massachusetts	Adopted	January 1, 2014
Michigan	Adopted	April 1, 2014
Minnesota	Adopted	January 1, 2014
Mississippi	Not adopted	NA
Missouri	Not adopted	NA
Montana	Adopted	January 1, 2016
Nebraska	Not adopted	NA
Nevada	Adopted	January 1, 2014
New Hampshire	Adopted	August 15, 2014
New Jersey	Adopted	January 1, 2014
New Mexico	Adopted	January 1, 2014
New York	Adopted	January 1, 2014
North Carolina	Not adopted	NA
North Dakota	Adopted	January 1, 2014
Ohio	Adopted	January 1, 2014
Oklahoma	Not adopted	NA
Oregon	Adopted	January 1, 2014
Pennsylvania	Adopted	January 1, 2015
Rhode Island	Adopted	January 1, 2014
South Carolina	Not adopted	NA
South Dakota	Not adopted	NA
Tennessee	Not adopted	NA
Texas	Not adopted	NA
Utah	Not adopted	NA
Vermont	Adopted	January 1, 2014
Virginia	Not adopted	NA
Washington	Adopted	January 1, 2014
West Virginia	Adopted	January 1, 2014
Wisconsin	Not adopted	NA
Wyoming	Not adopted	NA

Abbreviation: NA, not applicable.

^a^ States' decisions about adopting the Medicaid expansion are as of December 31, 2017.

^b^ Alaska is excluded from analyses because of substantial changes in the size and shape of counties during the study period.

### Covariates

Annual, county-level estimates for a range of sociodemographic characteristics were obtained from GeoLytics Inc to be used as covariates, including age (percentage aged 0-19, 20-24, 25-44, and 45-64 years); percentage male; percentages non-Hispanic white, non-Hispanic Black, and Hispanic; percentage of families living in poverty; median household income (per $10 000); percentage unemployed; population density (1000 residents per square mile); and overall mortality rate (per 1000 people). We also considered the presence of co-occurring state policies, which have been associated in prior research^19,20,21^ with changes in opioid-related harm, including prescription drug monitoring programs, overdose Good Samaritan laws, naloxone access laws, and medical marijuana laws. Information on these policies was derived from the Prescription Drug Abuse Policy System^22^ and from McClellan and colleagues^19^ and updated by us.

### Statistical Analysis

We examined the association between state Medicaid expansion status and county-level risk of fatal opioid overdoses overall and by class of opioid using Bayesian hierarchical Poisson models, with overdose deaths assumed to be distributed proportionally to the population of each county (aged ≥12 years). We introduced a 1-year lag between overdose rates and Medicaid expansion to address the possibility of temporal bias and to allow time for changes in Medicaid coverage, services, and related behaviors to materialize. Analyses with Medicaid expansion instead measured concurrently with overdose rates produced similar results (eTable 2 in the Supplement). Furthermore, because drug-specific overdose rates may be variously underestimated or overestimated among states^23^ and for comparison with prior research, we conducted a secondary analysis with all drug overdose deaths as the outcome.

In practice, our models compared overdose trends in counties within states that expanded Medicaid before vs after the expansion with trends in counties within nonexpansion states. Unlike conventional difference-in-difference methods, the Bayesian approach does not assume that trends in overdose deaths before Medicaid expansion were the same among counties within expansion and nonexpansion states. Instead, by incorporating county-level random intercepts and trends, along with state-level fixed effects, growth mixtures among counties within states that occurred during the study period and could bias effect estimates were explicitly modeled. We also included conditional autoregressive spatial random effects, which account for the lack of independence in spatially contiguous counties (ie, spatial autocorrelation) and minimize the influence of large outlying rates in low-population counties by allowing each area to borrow strength from neighboring areas. All models also included fixed and random effects by county for Medicaid expansion to account for local variation in policy implementation across counties within states. We modeled secular trends in overdose using fixed linear and quadratic time trends and included annual, county-level sociodemographic covariates measured concurrently with overdose and co-occurring state policies with 1-year time lags.

Analyses were implemented using the Integrated Nested Laplace Approximation method in R software, version 3.4.3 (R Project for Statistical Computing)^24^ from April 1, 2018, to July 31, 2019. Integrated nested Laplace approximation is an alternative to standard Markov chain Monte Carlo methods for estimating the integral of a posterior (probability) distribution. Whereas Markov chain Monte Carlo samples from the posterior distribution of model parameters, integrated nested Laplace approximation returns comparable approximations to the posterior marginals in considerably less time.^25,26^ Results are reported as median relative rates (RRs) from the posterior marginal distribution and 95% credible intervals (CrIs) indicating a range of values that is expected to contain the true RR with 95% probability (a Bayesian analogue of a standard CI).

## Results

**Figure 1.**
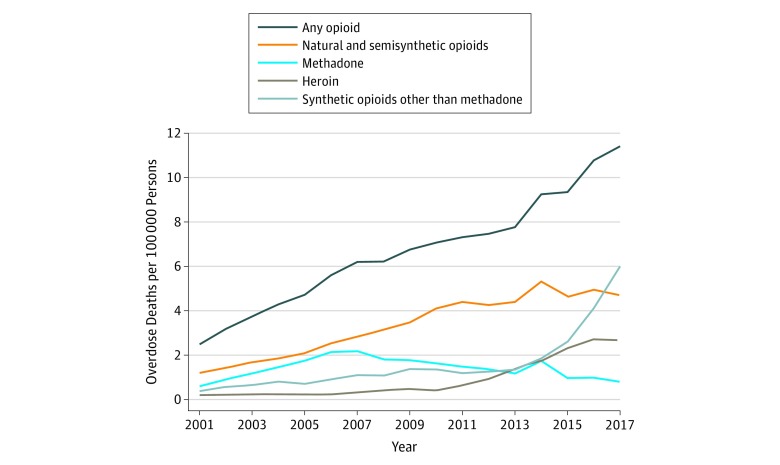
Opioid Deaths per 100 000 Persons

There was a total of 383 091 opioid overdose fatalities across observed US counties for the study period of January 1, 2001, through December 31, 2017, with a mean (SD) of 7.25 (27.45) deaths per county (range, 0-1145 deaths per county) (Table 2). The overall opioid mortality rate increased over time, from 2.49 deaths per 100 000 people in 2001 to 11.41 deaths per 100 000 in 2017 (Figure 1). Rates were generally higher in expansion states than in nonexpansion states (eFigure in the Supplement). Overdoses involving natural and semisynthetic opioids accounted for the largest share of all county-year opioid overdose deaths (40.9%), followed by those involving heroin (25.3%), synthetic opioids other than methadone (24.0%), and methadone (17.1%). By 2017, most opioid overdose deaths (59.9%) involved synthetic opioids other than methadone (eg, illicitly manufactured fentanyl).

**Table 2.** County-Level Fatal Opioid Overdoses and Sociodemographic Characteristics, United States, 2001-2017^a^

Characteristic	Mean (SD) [Range]	Mean Change for 2017 vs 2001
Opioid-related deaths		
No.	7.25 (27.45) [0-1145.00]	12.31
Rate, No./100 000 population	6.69 (13.80) [0-2083.33]	8.92
Natural or semisynthetic opioid–related deaths		
No.^b^	2.96 (10.71) [0-278.00]	3.55
Rate, No./100 000 population	3.36 (11.27) [0-2083.33]	3.49
Methadone-related deaths		
No.^b^	1.24 (4.27) [0-98.00]	0.56
Rate, No./100 000 population	1.42 (9.75) [0-2083.33]	0.20
Heroin-related deaths		
No.^b^	1.84 (11.08) [0-758.00]	4.43
Rate, No./100 000 population	0.91 (3.05) [0-75.30]	2.47
Synthetic opioid–related deaths		
No.^b^	1.74 (12.44) [0-687.00]	8.90
Rate, No./100 000 population	1.61 (4.80) [0-195.49]	5.61
Population aged ≥12 y, No.	82 415.89 (263 708.70) [34.00-8 649 898.00]	11 427.32
Age, %		
0-19 y	26.80 (4.34) [0-134.09]	−0.94
20-24 y	6.91 (1.20) [0-32.53]	0.59
25-44 y	25.06 (3.46) [0-124.04]	−2.27
45-64 y	25.05 (3.02) [0-127.15]	−0.23
Male, %	49.56 (2.18) [35.23-249.61]	−0.31
Race/ethnicity, %		
White	76.31 (20.35) [0-355.51]	−8.52
Black	8.89 (14.81) [0-91.74]	−0.36
Latinx	7.39 (12.99) [0-105.52]	1.93
Living in poverty, %	12.60 (6.59) [0-61.63]	3.37
Median household income per $10 000, $	4.54 (1.24) [1.27-34.90]	−0.18
Unemployed, %	6.96 (4.17) [0-67.28]	0.09
Population density, 1000 per square mile^c^	0.22 (1.25) [0-50.92]	0.02
Overall mortality rate, No./1000 residents	8.58 (3.69) [0-125.00]	0.62

^a^ Sample size was 3109 counties from 2001 to 2017 (52 853 county-years).

^b^ Deaths involving more than 1 class of opioid were included in the counts for each opioid subcategory.

^c^ The mean population density was 0.22 × 1000 or 220 per square mile.

**Figure 2.**
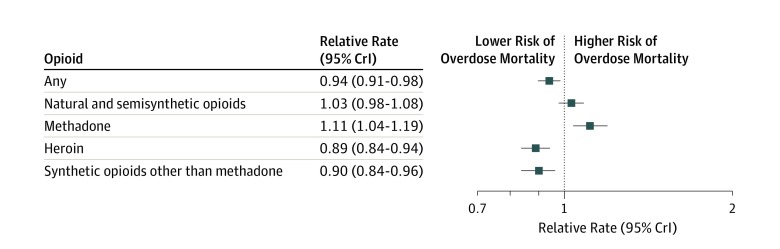
Estimated Associations of 1-Year Lagged Medicaid Expansion With Relative Rates of Opioid Overdose Deaths Overall and by Class of Opioid CrI indicates credible interval.

The estimated associations of 1-year lagged Medicaid expansion with RRs of opioid overdose deaths, overall and by class of opioid, are presented in Figure 2 (results for all model variables are in eTable 1 in the Supplement). Medicaid expansion was associated with lower risk of overdose mortality involving all opioids. Specifically, counties within states that expanded Medicaid had a 6% decreased rate of opioid overdose deaths after expansion compared with counties within states that did not expand Medicaid eligibility (RR, 0.94; 95% CrI, 0.91-0.98). In drug-specific analyses, counties within states that expanded Medicaid had an 11% decreased rate of fatal heroin overdoses (RR, 0.89; 95% CrI, 0.84-0.94) and a 10% decreased rate of overdose deaths involving synthetic opioids other than methadone (RR, 0.90; 95% CrI, 0.84-0.96) after the expansion compared with counties in nonexpansion states. In contrast, the expansion was associated with an 11% increased rate of methadone-involved overdose deaths (RR, 1.11; 95% CrI, 1.04-1.19). An association between Medicaid expansion and deaths involving natural and semisynthetic opioids was not well supported (RR, 1.03; 95% CrI, 0.98-1.08).

Consistent with previous research, our secondary analysis of overdose fatalities involving all drugs found that counties within states that expanded Medicaid had a 2% decreased rate of all drug overdose deaths after the expansion compared with those in nonexpansion states (RR, 0.98; 95% CrI, 0.96-1.00). Additional sensitivity analyses excluding 4 states with high levels of underreporting of specific drugs (ie, Alabama, Indiana, Louisiana, and Pennsylvania)^23^ produced substantively similar results as those in the primary analyses (eTable 2 in the Supplement).

## Discussion

In this nationwide, population-based study of the association of Medicaid expansion under the ACA with county-level rates of opioid overdose mortality, we found empirical support for adopting and sustaining health coverage expansions as a potential tool for reducing opioid overdose deaths in the United States. Consistent with prior analyses^16,27^ examining Medicaid expansion and mortality from other causes, we found decreased rates of opioid overdose deaths associated with the adoption of Medicaid expansion. In particular, given 82 228 opioid-related deaths from 2015 to 2017 in the 32 states that expanded Medicaid between 2014 and 2016, our findings suggest that these states would have had between 83 906 and 90 360 deaths in the absence of the expansion, implying that Medicaid expansion may have prevented between 1678 and 8132 deaths in these states during those years.

In analyses differentiated by class of opioid, we found a more substantial decreased risk associated with overdose deaths involving heroin and synthetic opioids other than methadone, which have been associated with continued increases in opioid-related deaths in recent years. These findings align with previous research that indicates that implementation of the ACA was associated with 40% decreased odds of being uninsured among persons with heroin use disorders, primarily because of Medicaid expansion, whereas no changes in insurance coverage were detected among persons with prescription OUDs.^28^ We also did not find support for an association between ACA-related Medicaid expansion and natural and semisynthetic opioid overdose mortality.

The observed association between Medicaid expansion and decreased total opioid overdose deaths and deaths involving heroin and synthetic opioids other than methadone is likely in part attributable to the ACA’s inclusion of mental health and SUD services as essential health benefits. Expanded Medicaid eligibility has substantially increased access to these services among the low-income population.^10,29^ Recent evidence demonstrates that compared with nonexpansion states, Medicaid expansion states experienced increases in overall prescriptions for, Medicaid-covered prescriptions for, and Medicaid spending on both MOUDs, particularly buprenorphine and naltrexone, and the opioid overdose reversal medication naloxone.^6,7,8,11,14,30,31,35^

Two prior studies^12,16^ have found associations between income eligibility expansions for Medicaid and reductions in SUD-related deaths, and a recent study^17^ assessed changes in opioid-related deaths in Medicaid expansion vs nonexpansion states. Whereas the last study^17^ found that Medicaid expansion was associated with larger increases in opioid overdose mortality, particularly in 2015 and 2016, analyses were conducted only at the state level. This approach may have masked within-state variation in the level and rate of growth of opioid overdoses, as well as differences in local policy implementation. To our knowledge, ours is the first study to quantify the association between ACA-related Medicaid expansion and opioid-related deaths at the county level.

Although the rate of methadone-related mortality is relatively low compared with other opioid classes, our finding that Medicaid expansion was associated with increased methadone overdose deaths deserves further investigation. At the individual level, treatment of OUD with methadone has been rigorously studied and found to be equally and, in some cases, more effective than other MOUDs in suppressing illicit opioid use, particularly heroin use, and retaining persons in treatment.^31,32^ On the basis of this evidence, in combination with our findings for heroin and synthetic opioids other than methadone, increased access to MOUDs likely not did not contribute to the observed increase in methadone mortality associated with Medicaid expansion. In contrast, past research has found high rates of methadone use to treat pain (rather than to treat OUD) among Medicaid beneficiaries and that the drug is disproportionately associated with overdose deaths among individuals in this population,^33,34^ underscoring the importance of ongoing local, state, and federal actions to address safety concerns associated with methadone for pain in tandem with Medicaid expansion.^7,8^

### Limitations

This study has limitations. First, we relied on *ICD-10* coding of death certificate data, which may not reliably identify the specific drugs involved in fatal overdoses and may lead to an underestimation or misclassification of opioid overdose mortality.^23^ However, a secondary analysis that examined overdose deaths involving all drugs and sensitivity analyses excluding states with high levels of underreporting of specific drugs produced similar results as those in our primary models. Second, we included deaths from opioid overdoses across the entire population, not just among Medicaid enrollees, which may understate the estimated outcomes of Medicaid expansion for those individuals most directly affected. Third, although we controlled for various county-level sociodemographic characteristics and state-level co-occurring policies, unmeasured confounding is still a possibility. Fourth, we did not examine the specific provisions of Medicaid expansion that may be associated with changes in opioid-related deaths (eg, state-level difference in Medicaid’s preferred drug lists). In addition, this study focused on the association of Medicaid expansion with fatal overdoses only. Future studies should consider the association of expansion with the spectrum of opioid-related harms, including prevention of SUD and nonfatal overdoses. Also, future studies should explicitly examine possible mediators and moderators of the association between Medicaid expansion and opioid overdose risk, including access to and use of OPRs, MOUDs, and naloxone; local SUD treatment capacity; and the extent to which the association of Medicaid expansion with overdoses varies by individual sociodemographic characteristics and contextual conditions.

## Conclusions

This study found that Medicaid expansion was associated with reductions in opioid overdose deaths, particularly deaths involving heroin and synthetic opioids other than methadone, but with increases in methadone-related mortality. These findings add to the emerging body of evidence that Medicaid expansion under the ACA may be a critical component of state efforts to address the continuing opioid overdose epidemic in the United States. As states invest more resources in such efforts, attention should be paid to the role that health coverage expansions can play in reducing opioid overdose mortality, potentially through greater access to MOUDs.

## Notes

Supplement. **eFigure.** Opioid-Related Overdose Deaths per 100,000 People in United States Counties, by State Medicaid Expansion Status, 2001-17 **eTable 1.** Relative Rates Associated With Medicaid Expansion and County Characteristics, Total Opioid-Related Overdose Deaths and by Class of Opioid **eTable 2.** Relative Rates Associated With Medicaid Expansion and County Characteristics, Total Opioid-Related Overdose Deaths, by Primary and Alternative Model Specifications
