# Supplementary material for: Association of Medicaid Expansion With Opioid Overdose Mortality in the United States
Source: JAMA Netw Open. 2020 Jan 10;3(1):e1919066. doi: 10.1001/jamanetworkopen.2019.19066 (PMC6991255; doi:10.1001/jamanetworkopen.2019.19066)
Supplement: Supplement. — eFigure. Opioid-Related Overdose Deaths per 100,000 People in United States Counties, by State Medicaid Expansion Status, 2001-17 eTable 1. Relative Rates Associated With Medicaid Expansion and County Characteristics, Total Opioid-Related Overdose Deaths and by Class of Opioid eTable 2. Relative Rates Associated With Medicaid Expansion and County Characteristics, Total Opioid-Related Overdose Deaths, by Primary and Alternative Model Specifications [file jamanetwopen-3-e1919066-s001.pdf]

Supplementary Online Content

Kravitz-Wirtz N, Davis CS, Ponicki WR, et al. Association of Medicaid expansion with opioid overdose mortality in the United States. *JAMA Netw Open*. 2020;3(1):e1919066. doi:10.1001/jamanetworkopen.2019.19066

eFigure. Opioid-Related Overdose Deaths per 100,000 People in United States Counties, by State Medicaid Expansion Status, 2001-17

eTable 1. Relative Rates Associated With Medicaid Expansion and County Characteristics, Total Opioid-Related Overdose Deaths and by Class of Opioid

eTable 2. Relative Rates Associated With Medicaid Expansion and County Characteristics, Total Opioid-Related Overdose Deaths, by Primary and Alternative Model Specifications

This supplementary material has been provided by the authors to give readers additional information about their work.

© 2020 Kravitz-Wirtz N et al. *JAMA Network Open.*

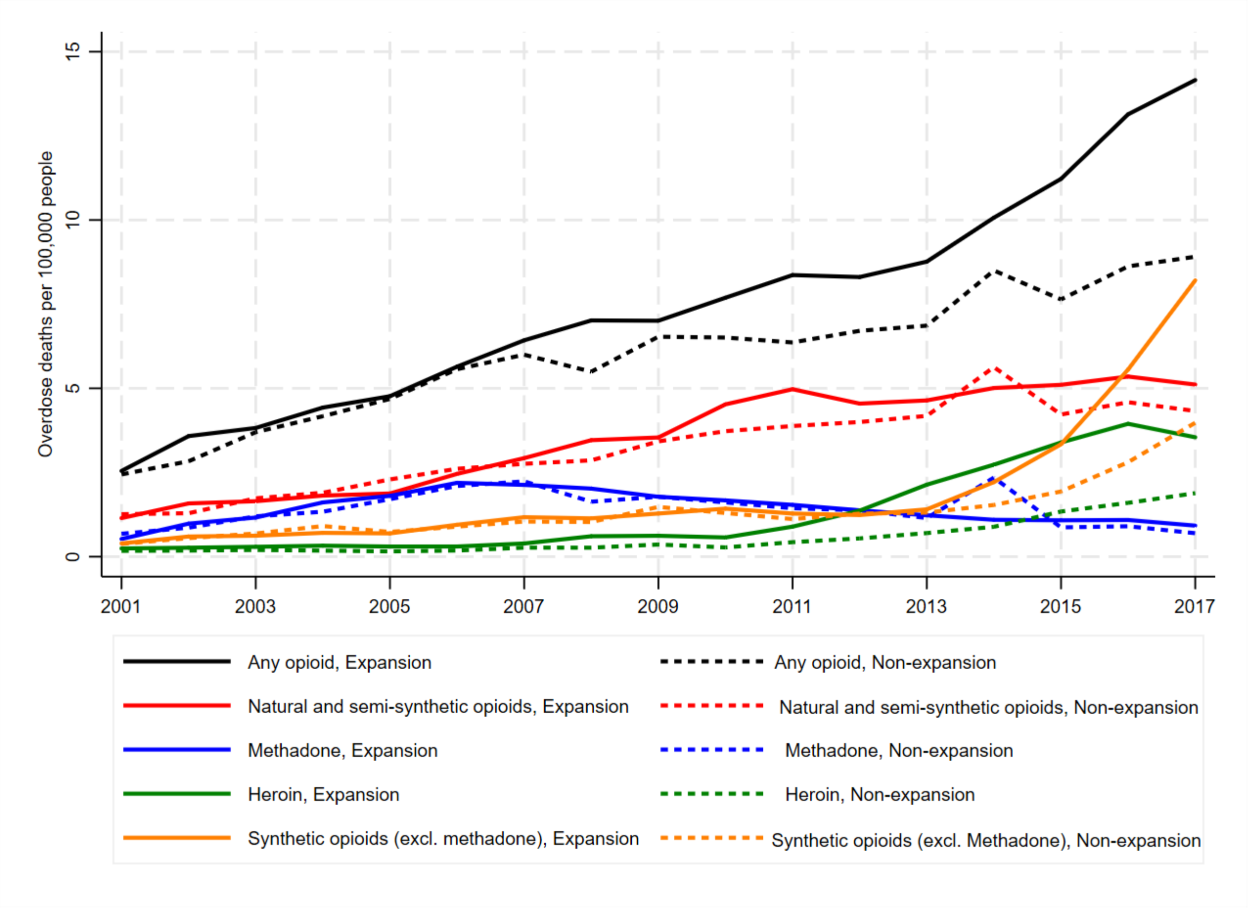

eFigure. Opioid-related overdose deaths per 100,000 people in United States counties, by state Medicaid expansion status, 2001-17

© 2020 Kravitz-Wirtz N et al. *JAMA Network Open.*

eTable 1. Relative rates associated with Medicaid expansion and county characteristics, total opioid-related overdose deaths and by class of opioid

	Any opioid		Natural and semi-synthetic opioids		Methadone		Heroin		Synthetic opioids (excl. Methadone)	
	RR	95% CI	RR	95% CI	RR	95% CI	RR	95% CI	RR	95% CI
Medicaid expansion	0.94	[0.91-0.98]	1.03	[0.98-1.08]	1.11	[1.04-1.19]	0.89	[0.84-0.94]	0.90	[0.84-0.96]
Time	1.08	[1.08-1.09]	1.20	[1.19-1.21]	1.29	[1.27-1.32]	1.25	[1.23-1.27]	0.87	[0.86-0.89]
Time squared	1.00	[1.00-1.00]	0.99	[0.99-0.99]	0.98	[0.98-0.99]	1.00	[1.00-1.00]	1.01	[1.01-1.01]
Overall mortality rate (per 1,000)	1.05	[1.05-1.06]	1.06	[1.05-1.06]	1.06	[1.06-1.07]	1.05	[1.04-1.06]	1.05	[1.05-1.06]
Proportion aged 0-19	0.99	[0.99-1.00]	1.00	[0.99-1.01]	1.00	[0.99-1.01]	1.05	[1.04-1.07]	0.99	[0.98-0.99]
Proportion aged 20-24	1.01	[1.00-1.02]	1.01	[1.00-1.03]	1.03	[1.01-1.05]	0.98	[0.95-1.01]	1.05	[1.03-1.07]
Proportion aged 25-44	1.03	[1.03-1.04]	1.01	[1.00-1.02]	1.03	[1.02-1.04]	1.06	[1.05-1.07]	1.04	[1.03-1.05]
Proportion aged 45-64	1.03	[1.02-1.04]	1.02	[1.01-1.03]	1.07	[1.05-1.08]	1.00	[0.98-1.02]	1.09	[1.07-1.10]
Proportion male	0.97	[0.96-0.98]	0.99	[0.98-1.00]	0.95	[0.93-0.96]	0.93	[0.91-0.94]	0.97	[0.95-0.98]
Proportion white	1.00	[1.00-1.00]	1.00	[1.00-1.00]	1.00	[1.00-1.01]	0.99	[0.99-1.00]	1.01	[1.01-1.02]
Proportion black	0.99	[0.99-1.00]	0.99	[0.99-1.00]	0.99	[0.98-0.99]	1.00	[1.00-1.01]	1.01	[1.00-1.01]
Proportion Latinx	1.01	[1.01-1.01]	1.00	[1.00-1.01]	1.01	[1.00-1.01]	0.99	[0.99-1.00]	1.02	[1.01-1.02]
Proportion families in poverty	0.99	[0.99-1.00]	1.00	[0.99-1.00]	1.01	[1.00-1.01]	0.99	[0.98-1.00]	0.97	[0.96-0.97]
Median HH income ($10,000s)	0.96	[0.94-0.97]	0.96	[0.94-0.98]	0.97	[0.95-0.99]	1.29	[1.26-1.33]	0.74	[0.72-0.76]
Proportion unemployed	1.00	[0.99-1.00]	1.01	[1.01-1.01]	0.98	[0.98-0.99]	0.98	[0.98-0.98]	0.99	[0.99-0.99]
Population density	1.04	[1.02-1.06]	1.03	[1.00-1.05]	1.04	[1.01-1.07]	1.05	[1.01-1.09]	1.03	[1.00-1.05]
Overdose Good Samaritan law	1.02	[1.00-1.04]	0.93	[0.90-0.96]	1.05	[1.00-1.11]	1.17	[1.13-1.22]	1.09	[1.05-1.14]
Naloxone access law	1.12	[1.10-1.15]	1.14	[1.10-1.17]	1.13	[1.08-1.19]	1.09	[1.05-1.13]	1.24	[1.19-1.29]
Medical marijuana law	1.21	[1.18-1.25]	1.05	[1.01-1.10]	1.22	[1.15-1.30]	0.88	[0.84-0.93]	1.55	[1.47-1.63]
Prescription drug monitoring program	0.95	[0.94-0.97]	0.96	[0.93-0.98]	0.89	[0.86-0.92]	1.14	[1.10-1.19]	0.88	[0.84-0.91]

Notes: Posterior results are from Bayesian hierarchical Poisson analyses of 3109 US counties over the years 2001-17 (total n=52,853). Raw Poisson coefficients and credible intervals have been exponentiated and are thus interpretable as relative rates. Additional adjustments include state-level fixed effects, county-level random intercepts and trends, county-level random effects for Medicaid expansion, and conditional autoregressive (CAR) random effects.

© 2020 Kravitz-Wirtz N et al. *JAMA Network Open.*

eTable 2. Relative rates associated with Medicaid expansion and county characteristics, total opioid-related overdose deaths, by primary and alternative model specifications

	Primary model		Medicaid expansion, No lag[a]		Excluding states with high under-reporting[b]	
	RR	95% CI	RR	95% CI	RR	95% CI
Medicaid expansion	0.94	[0.91-0.98]	0.93	[0.90-0.97]	0.91	[0.88-0.94]
Time	1.08	[1.08-1.09]	1.07	[1.06-1.08]	1.08	[1.07-1.09]
Time squared	1.00	[1.00-1.00]	1.00	[1.00-1.00]	1.00	[1.00-1.00]
Overall mortality rate (per 1,000)	1.05	[1.05-1.06]	1.05	[1.05-1.06]	1.05	[1.05-1.05]
Proportion aged 0-19	0.99	[0.99-1.00]	0.99	[0.99-1.00]	0.99	[0.99-1.00]
Proportion aged 20-24	1.01	[1.00-1.02]	1.01	[1.00-1.02]	1.02	[1.01-1.03]
Proportion aged 25-44	1.03	[1.03-1.04]	1.03	[1.02-1.04]	1.04	[1.03-1.05]
Proportion aged 45-64	1.03	[1.02-1.04]	1.03	[1.02-1.04]	1.04	[1.03-1.05]
Proportion male	0.97	[0.96-0.98]	0.97	[0.97-0.98]	0.97	[0.96-0.98]
Proportion white	1.00	[1.00-1.00]	1.00	[1.00-1.00]	1.00	[1.00-1.00]
Proportion black	0.99	[0.99-1.00]	0.99	[0.99-1.00]	0.99	[0.99-0.99]
Proportion Latinx	1.01	[1.01-1.01]	1.01	[1.01-1.01]	1.00	[1.00-1.01]
Proportion families in poverty	0.99	[0.99-1.00]	0.99	[0.99-1.00]	1.00	[0.99-1.00]
Median HH income ($10,000s)	0.96	[0.94-0.97]	0.94	[0.93-0.95]	0.95	[0.94-0.97]
Proportion unemployed	1.00	[0.99-1.00]	1.00	[0.99-1.00]	1.00	[1.00-1.00]
Population density	1.04	[1.02-1.06]	1.04	[1.02-1.06]	1.07	[1.05-1.09]
Overdose Good Samaritan law	1.02	[1.00-1.04]	1.05	[1.03-1.07]	1.02	[1.00-1.04]
Naloxone access law	1.12	[1.10-1.15]	1.12	[1.10-1.14]	1.12	[1.10-1.15]
Medical marijuana law	1.21	[1.18-1.25]	1.22	[1.19-1.25]	1.23	[1.20-1.26]
Prescription drug monitoring program	0.95	[0.94-0.97]	0.97	[0.96-0.99]	0.96	[0.95-0.98]

[a]Medicaid expansion status was measured concurrently with opioid overdose deaths (rather than with a 1-year lag)

[b]4 states with high levels of underreporting of specific drugs (Alabama, Indiana, Louisiana, and Pennsylvania) were excluded; total n=47,923

Notes: Posterior results are from Bayesian hierarchical Poisson analyses of 3109 US counties over the years 2001-17 (total n=52,853). Raw Poisson coefficients and credible intervals have been exponentiated and are thus interpretable as relative rates. Additional adjustments include state-level fixed effects, county-level random intercepts and trends, county-level random effects for Medicaid expansion, and conditional autoregressive (CAR) random effects.

© 2020 Kravitz-Wirtz N et al. *JAMA Network Open.*
